# Simultaneous Determination of Three Active Forms of Vitamin B12 In Situ Produced During Fermentation by LC-MS/MS

**DOI:** 10.3390/foods14020309

**Published:** 2025-01-17

**Authors:** Zhihao Fan, Yajie Li, Xia Fan, Pei Wang, Runqiang Yang, Chong Xie

**Affiliations:** Whole Grain Food Engineering Research Center, College of Food Science and Technology, Nanjing Agricultural University, Nanjing 210095, China; fanzhihao@stu.njau.edu.cn (Z.F.); yajieli@stu.njau.edu.cn (Y.L.); fanxia@njau.edu.cn (X.F.); wangpei@njau.edu.cn (P.W.); yangrq@njau.edu.cn (R.Y.)

**Keywords:** vitamin B12, LC-MS/MS, fermentation, rice bran, *Propionibacterium freudenreichii*

## Abstract

The in situ fortification of vitamin B12 (VB12) in foods through fermentation is an effective strategy to address the deficiency of this micronutrient, and precise monitoring of VB12 production is crucial for developing VB12-fortified functional foods. Liquid chromatography–tandem mass spectrometry (LC-MS/MS) is advantageous for analyzing trace substances in food due to its high sensitivity. In the present study, an LC-MS/MS method capable of rapidly and accurately quantifying three active forms of VB12, namely adenosylcobalamin (AdoCbl), methylcobalamin (MeCbl), hydroxocobalamin (OHCbl), in 8 min were developed. Meanwhile, the quantitative result of this method is not affected by pseudo-VB12 because the selected ion channels include fragments of active VB12. Maintaining light-shielding during extraction and purification is essential, as light exposure during the process can decrease the content of detected VB12 by about 30%. At last, the developed method was applied for the determination of VB12 in fermented rice bran and the cell mass of *Propionibacterium freudenreichii*. The results showed that AdoCbl was the predominant form of VB12 during fermentation, and the addition of cobalt did not influence the proportions of the three VB12 types. The present study reported a rapid and accurate method for the simultaneous determination of three active forms of VB12, which can effectively support the development of foods with VB12 fortification.

## 1. Introduction

Vitamin B12 (VB12) is an essential water-soluble micronutrient that plays a pivotal role within the human body, and its deficiency may result in various health issues, such as numbness, depression, dementia, and megaloblastic anemia [1,2]. The recommended daily intake of VB12 in adults is 2.4 μg/day, and animal-based foods are the main dietary sources of this vitamin because it is virtually absent in natural plant-based foods [3]. Currently, some populations in vast regions across the world are still suffering from vitamin B12 deficiency caused by the inadequate intake of animal-based products due to various reasons, such as dietary habits and economics [4]. Meanwhile, the trend of replacing animal-based foods with plant-based foods will further decrease the VB12 intake among the general population. Therefore, the development of plant-derived foods rich in VB12 has become a popular research topic.

VB12 has the most complex chemical structure among all vitamins, and it is also known as cobalamin because it is characterized by a corrin ring structure centered on a cobalt ion with upper and lower ligands [5]. The lower ligand of VB12 is dimethylbenzimidazole (DMBI) moiety, which contains a benzimidazole ring with two methyl groups and one of the nitrogen atoms within the benzimidazole ring attached to the cobalt. Four groups, namely adenosyl, methyl, hydroxyl, and cyanide, can be present in the upper ligands, which form four distinct forms of VB12, namely adenosylcobalamin (AdoCbl), methylcobalamin (MeCbl), hydroxocobalamin (OHCbl), and cyanocobalamin (CNCbl). Among them, AdoCbl and MeCbl are two biologically active forms, and cyanocobalamin is the synthesized form that has been widely utilized due to its relatively high stability [3].

The fortification of foods with micronutrients is an effective way of overcoming micronutrient deficiencies. Due to the complex structure of VB12 molecules, their chemical synthesis is extremely difficult and costly. Therefore, VB12 is typically produced industrially through microbial fermentation [6]. The VB12 obtained through fermentation requires further separation, purification, and cyanidation before it can be used in food. On the contrary, the in situ production of VB12 in raw materials through microbial fermentation can eliminate these steps, making the process simpler and more environmentally friendly. Among them, *Propionibacterium freudenreichii* is a food-grade strain with high VB12 productivity and has been used in the fermentation of various foods to produce VB12, including breads [7], tempeh [8] and soy whey [9].

Accurate monitoring of VB12 production is essential for the development of functional foods through fermentation. Traditionally, VB12 is quantified by microbiological method, but subsequent research showed it cannot distinguish active VB12 and pseudo-B12, which is a VB12-like compound with low bioavailability, so the detection of VB12 currently employs methods such as liquid chromatography [10,11]. However, because VB12 has high photosensitivity, low content in food, and susceptibility to interference from other substances, complicated pretreatments are required before chromatographic detection, including purification and cyanidation. The purification process usually takes several hours and requires expensive purification columns, which incurs considerable cost and time. Although the cyanidation process can improve the photostability of VB12, utilization of cyanides can lead to environmental pollution and potential safety risks. Meanwhile, current studies showed different forms of VB12 may have different effects on microbiota and human health [12]. However, the cyanidation process converts all the VB12 forms into CNCbl, so it is impossible to quantify the exact content of AdoCbl, MeCbl, or OHCbl. Therefore, developing methods for the simultaneous determination of different natural forms of VB12 in fermented foods is very important.

Liquid chromatography–tandem mass spectrometry (LC-MS/MS) offers high sensitivity, making it an advantageous technique for analyzing trace substances in food. Meanwhile, by choosing specific ion pairs for monitoring, it is possible to distinguish structurally similar substances with high specificity. Therefore, this study aims to establish an LC-MS/MS method for the simultaneous determination of three active VB12 forms with high efficiency and precision. Additionally, the impact of light exposure during the operation process on the detection results has also been evaluated, and a case study of its application for the determination of VB12 in situ produced by *P. freudenreichii* in food material was conducted.

## 2. Materials and Methods

### 2.1. Materials and Reagents

Rice bran was obtained by milling brown rice, and the detailed information of the bran can be found in the previous study [13]. Bacteria used in this study were *P. freudenreichii* CICC 10019 and *Limosilactobacillus reuteri* DSM 20016, which both were obtained from the China Center of Industrial Culture Collection (CICC). Acetonitrile (HPLC grade) was obtained from Guanghua Sci-Tech Co., Ltd. (Shantou, China). Formic acid (LC-MS grade) and α-amylase were purchased from Macklin (Shanghai, China). Methanol (LC-MS grade) was provided by Supelco (Burlington, MA, USA). Three cobalamin standards, namely OHCbl, MeCbl, and AdoCbl, were purchased from Leyan Technologies Co., Ltd. (Shanghai, China), Yuanye Bio-Technology Co., Ltd. (Shanghai, China), and Weishi Reagent Group Co., Ltd. (Wuhan, China), respectively.

### 2.2. Activation of Bacteria and Fermentation of Rice Bran

*P. freudenreichii* and *L. reuteri* were cryopreserved at −80 °C in 25% glycerol (*v*/*v*). The *P. freudenreichii* culture was extracted from the cryopreservation vial and streaked onto the yeast extract and lactate (YEL) plate [14], followed by anaerobic incubation at a temperature of 30 °C for a duration of four to five days. Subsequently, individual colonies were isolated from the agar plates and transferred into a YEL broth for incubation (48 h at 30 °C). *L. reuteri* was activated on Man, Rogosa, and Sharpe (MRS) agar plates (Shengsi Bio-Technology Co., Ltd., Shanghai, China) at 30 °C for two days. Thereafter, individual colonies from the MRS agar plates were isolated and inoculated in MRS broth at 30 °C for 24 h. Cultures were harvested by centrifugation (6000× *g*, 10 min) and resuspended in sterile water. The obtained cell cultures were mixed with rice bran (8.0–8.5 log CFU/g), and the fermentation was conducted in a shaking incubator (30 °C, 200 rpm) for 3 days.

### 2.3. Extraction of VB12

For bacterial cell mass samples, VB12 was extracted as described by Chamlagain et al. [11] after minor modifications [11]. Briefly, the cell mass obtained by centrifugation (6000× *g*, 10 min) was weighed and suspended in 10 mL of extraction buffer (8.3 mM sodium hydroxide and 20.7 mM acetic acid, pH 4.5). The suspension was vortexed and then subjected to extraction in a boiling water bath. After 15 min, the tubes were cooled in an ice bath, followed by centrifugation at 6900× *g* for 10 min. The residue was resuspended in 5 mL of extraction buffer (pH 4.5) and centrifuged again. The supernatants from two instances of centrifugation were combined, and their volumes were adjusted to 25 mL with the same buffer. At last, the supernatants were filtered through a 0.22 μm syringe filter and stored at −20 °C before analysis or further purification.

For fermented bran samples, VB12 was extracted as described by Xie et al. [15] after minor modifications. Briefly, 3 g of sample was weighed and mixed with 15 mL of extraction buffer (pH 4.5) and 300 μL of α-amylase (50 mg/mL). The mixture was then incubated in a water bath at 37 °C for 30 min, followed by heating in boiling water for 15 min. Afterward, the samples were centrifuged (6900× *g*) at 4 °C for 20 min. The supernatants were collected, and the residues were resuspended in 5 mL of buffer and centrifuged again. After combining the supernatants, the final volume was adjusted to 25 mL with the buffer and filtered through a 0.22 μm syringe filter before analysis or further purification.

### 2.4. Purification of VB12

In the purification step, two types of purification columns were applied, namely Sep-Pak C18 solid-phase extraction column (Waters, Milford, MA, USA) and Easi-Extract VB12 immunoaffinity column (R-Biopharma, Glasgow, UK). Purification of VB12 with solid-phase extraction and VB12 immunoaffinity column columns were performed according to Zhu et al. [16] and Chamlagain et al. [11], respectively, with minor modifications. After purification, the collected eluates were evaporated using a nitrogen flow at 37 °C, and then the residues were dissolved in 500 µL ultrapure water before LC-MS/MS testing.

### 2.5. Determination of VB12 by LC-MS/MS and Validation of the Method

The analysis of VB12 was conducted using an LC–MS/MS system (Triple Quad 5500, AB Sciex; Framingham, MA, USA) following a method adapted from the literature [17]. Separation was achieved on a Kinetex 2.6 μm F5 100A LC column (2.1 mm × 100 mm, Phenomenex, Torrance, CA, USA) with water (mobile phase A) and acetonitrile (mobile phase B), both contained 50 mM formic acid, as the mobile phases. The flow rate was set at 0.0050 mL/min during the 0 to 2 min and 0.2500 mL/min during the 3 to 8 min. The gradient program was set as follows: 0.0–2.0 min, 90% A and 10% B; 3.0–4.5 min, 30% A and 70% B; 4.6–6.0 min, 0% A and 100% B; 6.1–8.0 min, 90% A and 10% B. The injection volume was 1 μL with the column temperature set at 35 °C, and the injector temperature maintained at 4 °C. LC-MS/MS was operated in electrospray ionization (ESI) positive with multiple reaction monitoring (MRM) scan type. The collision and desolvation of nitrogen gas were produced by a nitrogen generator (NiGen LCMS 40-1, Claind, Lenno, Italy). The standards of OHCbl, MeCbl, and AdoCbl were dissolved in ultrapure water and filtered through syringe filter (0.22 μm) to prepare standard solutions. The individual calibration curves were generated based on different levels of concentrations from 0.1 to 400.0 ng/mL. The LC-MS/MS method was evaluated for the linearity of the calibration curve, as well as for the limit of detection (LOD: signal-to-noise ratio, S/N = 3) and the limit of quantitation (LOQ, set as three times the LOD), using three cobalamin standards solutions for VB12.

### 2.6. Determination of VB12 by Enzyme-Linked Immunosorbent Assay (ELISA)

The VB12 ELISA kit was purchased from Enzyme-linked Biotechnology Co., Ltd. (Shanghai, China). The samples were pretreated as described in Section 2.3, and the VB12 content was determined according to the manual of the kit. The sample standard and detection antibody, labeled with horseradish peroxidase (HRP), were added to microwells that had been pre-coated with VB12 antibodies. The samples were then incubated and subjected to thorough washing. The substrate, 3,3′,5,5′-Tetramethylbenzidine (TMB), was used for color development. TMB was converted to a blue color under the catalysis of peroxidase and further converted to yellow under acidic conditions. The intensity of the color was found to be positively correlated with the VB12 concentration in the sample. Absorbance (OD value) was measured at a wavelength of 450 nm using a microplate reader, from which the VB12 concentration in the sample was calculated.

### 2.7. Statistical Analysis

The results were expressed as mean values with standard deviations from three replicates. Statistical analysis was carried out by SPSS 13.0 for Windows (IBM Corporation, New York, NY, USA). Significant differences among the samples were determined using one-way analysis of variance (ANOVA) or Fisher’s F-test at a significance level of *p* < 0.05. The LC–MS/MS data acquisition and processing were carried out using Analyst 1.5.1 software (AB Sciex) and SCIEX 1.7.0 software (AB Sciex).

## 3. Results and Discussion

### 3.1. Method Development and Validation

Up to now, fermented foods with in situ-produced VB12 have been considered a promising way of overcoming the deficiency of this micronutrient [18]. Although various VB12 quantitative methods have been developed, including microbiological assay [19], ELISA [20], HPLC [21], and inductively coupled plasma-mass spectrometry (ICP-MS) [22], their utilization in fermented foods for VB12 determination is limited by issues such as high detection limits, long processing times, high costs, and inadequate differentiation between different forms of VB12. In addition, current methods primarily convert all cobalamins into CNCbl to measure total VB12, whether it is for the detection of VB12 alone [23,24] or for the detection of B-vitamins [25,26], thereby cannot measure the contents of three natural cobalamins. In recent years, there have also been some reports about the determination of active cobalamin in animal plasma or food supplements [27,28]. However, complex preparation processes, such as online solid phase extraction, are still necessary to ensure high sensitivity in these samples that exhibit relatively low interference from impurities. Therefore, we aim to develop an LC-MS/MS method that can quickly and accurately quantify the three active forms of VB12 in more complex systems, such as fermented food.

**Figure 1:**
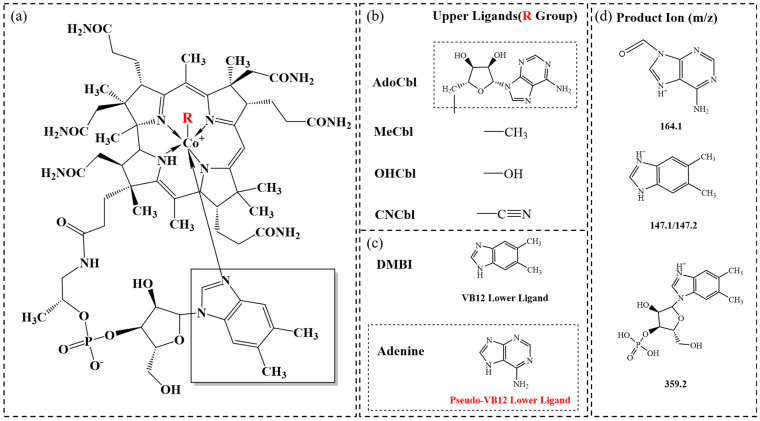
(**a**) Molecular structure of VB12; (**b**) upper ligands of VB12; (**c**) lower ligand of VB12 and pseudo-VB12; (**d**) ion fragments of three VB12 forms in this study.

In this study, mass spectrometric parameters were optimized by infusing 400 ng/mL solutions of each cobalamin at a rate of 7.0 μL/min via a syringe pump. For the tandem MS measurement, the following precursor ions were selected: *m*/*z* 527 ([M+3H]^3+^) for AdoCbl, *m*/*z* 664.9 ([M+H]^2+^) for OHCbl, and *m*/*z* 672.9 ([M+2H]^2+^) for MeCbl. As illustrated in Figure 1d, the molecular structure of fragment ions demonstrates the fragment ion at *m*/*z* 147.1/147.2, corresponding to the protonated adduct of DMBI. The ion at *m*/*z* 359.2 consists of the protonated adduct of the ribose-phosphate-benzimidazole moiety. The ion at *m*/*z* 164.1, observed exclusively for AdoCbl, is attributed to the protonated 6-amino-9H-purine-9-carbaldehyde [17]. One daughter ion was chosen for MeCbl, and two daughter ions were chosen for OHCbl and AdoCbl as the product ion in MRM scan mode.

**Figure 2:**
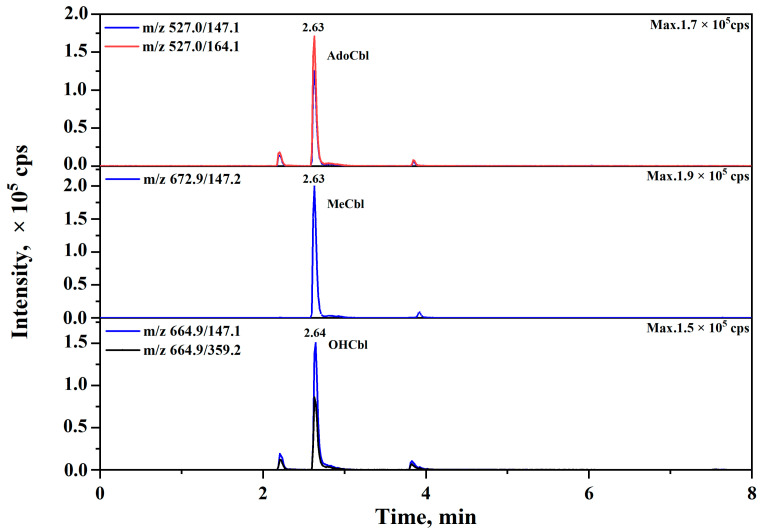
The ion chromatograms of the mixed standard solution of three cobalamins under optimized conditions.

The optimal values of DP and CE, as shown in Table 1, for each precursor-to-product transition were determined by monitoring the most intense MS response in MRM scan mode. A dwell time of 100 ms was employed for each MRM transition within a single retention time window. Three cobalamins had a retention time from 2.63 min to 2.65 min (Figure 2), and an external calibration curve with great linearity (R^2^ > 0.99) was achieved for each type of product ion (Table 1), by eluting with a proper linear gradient (Supplementary Figure S1). Notably, even though concentrations of VB12 were lower than 1 ng/mL, distinct peaks, which were significantly higher than the background noise, can still be observed in the chromatographs (Supplementary Figure S2) with a high precision (Supplementary Table S1). At last, an LC-MS/MS method that can simultaneously determine three cobalamins in 8 min was established with LODs at ca. 0.01 ng/mL for three forms of VB12 (Table 1). The LODs of the current LC-MS/MS method are lower than other HPLC methods, which usually have a LOD at levels of nanograms per gram, such as the high-performance liquid chromatography–inductively coupled plasma mass spectrometry (HPLC-ICP-MS) method with a LOD at 0.63 ng/g [29].

### 3.2. Distinguishing of VB12 and Pseudo-VB12

**Figure 3:**
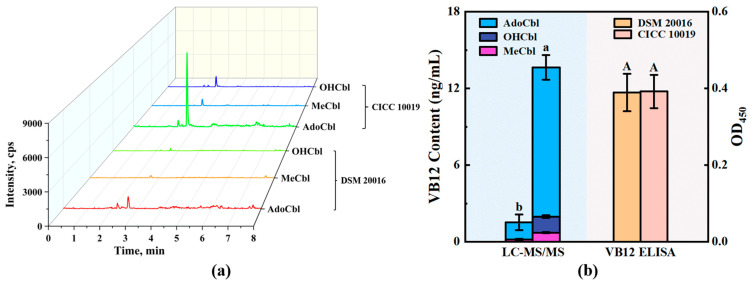
(**a**) Three types of cobalamin chromatograms in cell mass of *L. reuteri* DSM 20016 and *P. freudenreichii* CICC 10019; (**b**) VB12 contents of *L. reuteri* DSM 20016 and *P. freudenreichii* CICC 10019 quantified by two detection methods. Different letters indicate significant differences (*p* < 0.05).

When the lower ligand of VB12 attaches to groups other than DMBI, such as adenine, it is referred to as pseudo-VB12 (Figure 1c) [30]. Although some microorganisms can use pseudo-VB12 as a cofactor in their metabolism, it has an extremely low absorption rate in mammals [31]. *L. reuteri* DSM 20016, a well-studied strain of lactic acid bacterium, primarily produces pseudo-VB12 due to the absence of bluB, the necessary gene for the de novo biosynthesis of DMBI [30]. Thus, *L. reuteri* DSM 20016 was used as the source of pseudo-VB12 to test whether our established method has the ability to distinguish between VB12 and pseudo-VB12. As shown in Figure 3a, in the sample of *L. reuteri* cell mass, only a small peak of AdoCbl was observed, and there was almost no peak in OHCbl and MeCbl. In comparison, peaks representing three forms of VB12 were present in the sample of *P. freudenreichii*. Accordingly, LC-MS/MS quantification showed OHCbl was not detected in DSM 20016, and MeCbl as well as AdoCbl were detected only in very low levels (Figure 3b). However, ELISA analysis showed there was no significant difference (*p* < 0.05) between the VB12 contents in samples of *L. reuteri* and *P. freudenreichii* (Figure 3b), which indicates that the ELISA Kit used in this study cannot distinguish between VB12 and the pseudo-VB12 produced by *L. reuteri*. In contrast, the quantitative result of LC-MS/MS is not affected by pseudo-VB12 because the selected ion channels include fragments of DMBI (Table 1), the lower ligand of active VB12. Not only fermented foods but also some natural foods have also been found to contain high levels of pseudo-VB12, such as spirulina [32]. These LC-MS/MS methods can also be used to determine the content of active VB12 in these natural foods, which can help individuals with VB12 deficiency to more accurately choose reliable sources of this nutrient.

### 3.3. Light Stability

**Figure 4:**
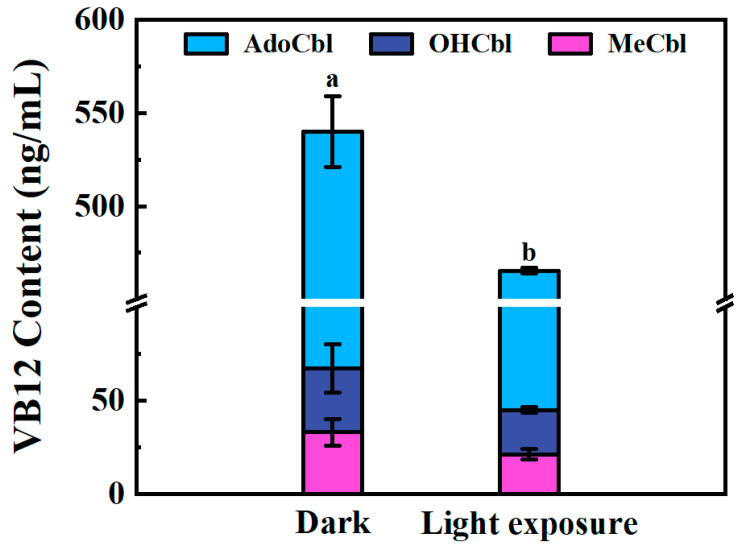
VB12 contents of samples that were treated in dark (control) or treated with light exposure. Different letters indicate significant differences (*p* < 0.05).

All vitamin B12 forms, especially MeCbl and AdoCbl, are susceptible to photodegradation [33]. The photolysis of the Co-methyl bond in MeCbl is found to generate Co (II)-cobalamin and a methyl radical [34]. During the photolysis of AdoCbl in the presence of oxygen, the 5′-deoxyadenosyl radical is rapidly reacted to form 5′-peroxyadenosine, which is subsequently decomposed to adenosine-5′-aldehyde and minor quantities of adenosine and adenine [35]. Under anaerobic conditions, cob(II) and 5′,8-cycloadenosine are primarily produced through the cyclization of the 5′-deoxyadenosyl radical [36,37,38]. The second major photolysis product of both MeCbl and AdoCbl, cob(II), is observed to remain stable under anaerobic conditions but undergoes rapid oxidation to aquocobalamin in the presence of oxygen, a reaction that is further enhanced by 5′-peroxyadenosine [35,39]. At physiological pH and under UVA radiation, OHCbl is shown to exhibit the highest stability among three forms of natural cobalamins, while AdoCbl and MeCbl are converted to OHCbl within seconds of UVA exposure [40]. In order to investigate the stability of the three cobalamins under light conditions during extraction and purification, the same samples that had been processed in a dark room and a room with natural light exposure were compared. As shown in Figure 4, when compared with the control sample, the total content of VB12 under the light was significantly decreased. More specifically, AdoCbl, OHCbl, and MeCbl decreased by 11.02%, 31.08%, and 35.73%, respectively (Figure 4). However, whether they transformed into each other or interacted during the degradation process was not studied in this work. In the future, high-resolution mass spectrometry can be used to further identify intermediate products at different times during the photodegradation process. This will help explore the degradation mechanisms of different forms of VB12, thereby exploring new methods of improving their stability. The current results showed it is crucial to keep the entire extraction and purification process as light-shielded as possible to ensure the accuracy of quantification. Moreover, for industries producing natural VB12-containing food, it is also important to make a relatively dark environment during production, packaging and transportation to guarantee the VB12 content does not decrease significantly.

### 3.4. Application of Detection Methods

Rice bran is the by-product of rice processing with abundant nutrients and bioactive components [41]. Previous studies showed rice bran produced the highest level of VB12 among 11 kinds of grain materials during fermentation with *P. freudenreichii* [42], and using fermented rice bran as a partial substitute for flour can produce bread with 7.6–7.9 μg/100 g (in fresh weight) VB12 without affecting the flavor and texture [7]. Considering the extremely complex composition of rice bran and its potential to be a raw material for producing VB12-fortified food, this material was chosen to test the feasibility of our detection method. Marley et al. [43] developed procedures for the determination of low levels of VB12 by HPLC with UV, in which an immunoaffinity purification column was required. In another study, a general-purpose column (Sep-Pak C18) was also used for VB12 purification and concentration [16]. In this study, VB12 contents in fermented rice bran treated with the general-purpose column and VB12 immunoaffinity purification column and without purification were compared.

**Figure 5:**
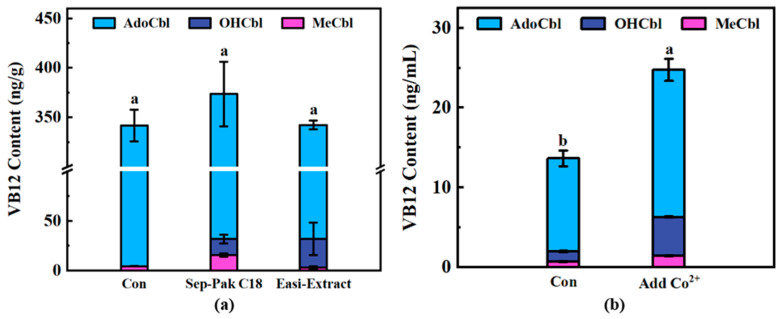
(**a**) VB12 content in fermented rice bran; Con, Sep-Pak C18, and Easi-Extract stand for samples with non-purification or purified with Sep-Pak C18 or Easi-Extract column. (**b**) VB12 content in cell mass with (Add Co^2+^) or without (Con) cobalt ions addition. Different letters indicate significant differences (*p* < 0.05).

As shown in Figure 5a, there was no significant difference in the content of total VB12 (*p* < 0.05) with or without purification, but there was a significant difference in the content of different forms of cobalamin (*p* < 0.05). Notably, OHCbl was not detected in unpurified fermented rice bran but was quantified in samples treated with two columns. Considering the processes were conducted in the dark, it is unlikely that photolysis was the cause. Rather, the content of OHCbl in the fermented rice bran may be too low to allow for quantification without purification and concentration. AdoCbl was the predominant form of VB12 in the fermented bran and there was no significant difference in the content among the treatments, regardless of whether purification was performed or not. This suggested that when the measured content is sufficiently high, the method can be used directly for determination without purification, which can save a lot of time and cost. However, for accurate quantification of the various VB12 contents, it is still preferable to process the samples through a purification column. There was no significant difference in the total VB12 content between the two types of purification columns, but there were differences in the contents of MeCbl and HOCbl. The reasons for this situation require further investigation. Additionally, The LC-MS/MS method utilized in the current study was specifically applied for the quantification of VB12 in rice bran and cell mass. Its applicability to other food matrices or fermented products remains unexplored.

In the synthesis of VB12 by *P. freudenreichii*, cobalt is involved as an electron donor in activating methylation at the C-1 position and participates in the subsequent multi-step reaction that promotes the structural rearrangement of the central corrin ring, which plays a key role in the synthesis of VB12 [44]. The addition of cobalt chloride in the durum flour fermentation was found to potentially result in a significant increase in VB12 yield [45]. However, it was not clear if the addition of cobalt could affect the respective proportions of the three types of VB12. Therefore, the profiles of VB12 in the cell mass with or without the addition of cobalt chloride were analyzed in the present study. The results showed that the addition of cobalt significantly increased the production of VB12 during fermentation, but there was no significant change in the respective proportions of the three types of VB12 (Figure 5b). Similar to the results of rice bran fermentation, AdoCbl was the predominant form of VB12 in the cell mass that harvested from YEL broth, which may be because it is the ultimate product during biosynthetic pathway of VB12 in microorganisms and participates in various biochemical processes by functioning as a coenzyme for many important enzymatic reactions [6].

## 4. Conclusions

In conclusion, the current study has successfully developed an LC-MS/MS method capable of rapidly and accurately quantifying three forms of VB12. Notably, the quantitative accuracy of this method remains unaffected by pseudo-VB12 due to the inclusion of ion channels that specifically target fragments of active VB12. It is crucial to maintain light-shielding conditions during the extraction and purification processes, as exposure to light can result in a significant decrease (approximately 30%) of VB12 content. By application of the developed method in determining VB12 in rice bran, it was showed when the measured content is sufficiently high, the method can be utilized directly for determination without the need for purification, thereby saving considerable time and cost. Furthermore, AdoCbl was the predominant form of VB12 during the fermentation of *P. freudenreichii*, and the addition of cobalt did not alter the proportions of the three VB12 types.

The current work only applied the LC-MS/MS method for quantification of VB12 in rice bran and cell mass; its utilization in other food matrices or fermented products has not been explored. Future studies can expand the application of the LC-MS/MS method to a wider range of biological samples to validate the robustness and versatility of the method. Meanwhile, AdoCbl was found to be the predominant form during the fermentation of *P. freudenreichii*. It is worth further investigation to determine whether this phenomenon is influenced by fermentation conditions, growth stages, metabolic substrates, and strains. This can help better explain the role of VB12 in the metabolism of microorganisms.

## Figures and Tables

**Table 1:** Composite MRM parameters of three cobalamin standards, linear ranges, regression equations, and correlation coefficients.

**Analytes**	**MRM Transition**	**RT (min)**	**DP (V)**	**CE (V)**	**Linear Range** **(ng/mL)**	**Limit of Detection**
OHCbl	664.9 → 147.1	2.65	125.0	66.0	0.10–400	0.01 ng/mL
	664.9 → 359.2 ^a^	2.65	100.0	32.0	0.10–400	
AdoCbl	527.0 → 147.1 ^a^	2.63	52.5	74.0	0.50–400	0.01 ng/mL
	527.0 → 164.1	2.63	64.0	60.0	0.50–400	
MeCbl	672.9 → 147.2 ^a^	2.63	60.0	20.5	0.50–400	0.01 ng/mL

^a^ quantifier transition. RT: retention time. DP: declustering potential. CE: collision energy.

## Data Availability

The original contributions presented in the study are included in the article, further inquiries can be directed to the corresponding author.

## Supplementary Materials

The following supporting information can be downloaded at: https://www.mdpi.com/article/10.3390/foods14020309/s1, Figure S1 Calibration curves of three VB12 forms. Figure S2 Chromatographs of three VB12 standards in the lowest content. Table S1. The repeatability test of the method.

## References

[1] Nielsen M.J., Rasmussen M.R., Andersen C.B.F., Nexø E., Moestrup S.K. (2012). Vitamin B12 transport from food to the body’s cells—A sophisticated, multistep pathway. Nat. Rev. Gastroenterol. Hepatol..

[2] Li K., Wang C., Wang Y., Fu L., Zhang N. (2023). Future foods, dietary factors and healthspan. J. Future Foods.

[3] Martens J.H., Barg H., Warren M., Jahn D. (2002). Microbial production of vitamin B12. Appl. Microbiol. Biotechnol..

[4] Sobczyńska-Malefora A., Delvin E., McCaddon A., Ahmadi K.R., Harrington D.J. (2021). Vitamin B12 status in health and disease: A critical review. Diagnosis of deficiency and insufficiency—Clinical and laboratory pitfalls. Crit. Rev. Clin. Lab. Sci..

[5] O’Leary F., Samman S. (2010). Vitamin B12 in health and disease. Nutrients.

[6] Fang H., Kang J., Zhang D. (2017). Microbial production of vitamin B(12): A review and future perspectives. Microb. Cell Factories.

[7] Wang Y., Xie C., Pulkkinen M., Edelmann M., Chamlagain B., Coda R., Sandell M.A., Piironen V., Maina N.H., Katina K.J.L. (2022). In situ production of vitamin B12 and dextran in soya flour and rice bran: A tool to improve flavour and texture of B12-fortified bread. LWT.

[8] Signorini C., Carpen A., Coletto L., Borgonovo G., Galanti E., Capraro J., Magni C., Abate A., Johnson S.K., Duranti M. (2018). Enhanced vitamin B12 production in an innovative lupin tempeh is due to synergic effects of Rhizopus and Propionibacterium in cofermentation. Int. J. Food Sci. Nutr..

[9] Tindjau R., Chua J.Y., Liu S.Q. (2024). Co-culturing *Propionibacterium freudenreichii* and *Bifidobacterium animalis* subsp. lactis improves short-chain fatty acids and vitamin B(12) contents in soy whey. Food Microbiol..

[10] Tsiminis G., Schartner E.P., Brooks J.L., Hutchinson M.R. (2017). Measuring and tracking vitamin B12: A review of current methods with a focus on optical spectroscopy. Appl. Spectrosc. Rev..

[11] Chamlagain B., Edelmann M., Kariluoto S., Ollilainen V., Piironen V. (2015). Ultra-high performance liquid chromatographic and mass spectrometric analysis of active vitamin B12 in cells of Propionibacterium and fermented cereal matrices. Food Chem..

[12] Guetterman H.M., Huey S.L., Knight R., Fox A.M., Mehta S., Finkelstein J.L. (2022). Vitamin B-12 and the Gastrointestinal Microbiome: A Systematic Review. Adv. Nutr. (Bethesda Md.).

[13] Xie C., Yuan R., Su L., Li D., Zhang C., Yin Y., Wang P., Yang R. (2024). Improving nutritional and sensory properties of rice bran by germination and solid-state fermentation with fungi. Food Biosci..

[14] Malik A.C., Reinbold G.W., Vedamuthu E.R. (1968). An evaluation of the taxonomy of Propionibacterium. Can. J. Microbiol..

[15] Xie C., Coda R., Chamlagain B., Varmanen P., Piironen V., Katina K. (2019). Co-fermentation of *Propionibacterium freudenreichii* and *Lactobacillus brevis* in Wheat Bran for in situ Production of Vitamin B12. Front. Microbiol..

[16] Zhu X., Xia Y., Wang H., Shi L., Yin H., Gu M., Yan F. (2023). PM2.5 induced neurotoxicity through unbalancing vitamin B12 metabolism by gut microbiota disturbance. Gut Microbes.

[17] Perez-Fernandez V., Gentili A., Martinelli A., Caretti F., Curini R. (2016). Evaluation of oxidized buckypaper as material for the solid phase extraction of cobalamins from milk: Its efficacy as individual and support sorbent of a hydrophilic-lipophilic balance copolymer. J. Chromatogr. A.

[18] Gomes Soares M., Bevilaqua G.C., Marcondes Tassi É.M., Reolon Schmidt V.C. (2023). Fermented foods and beverages: A potential in situ vitamin B12 biofortification—A literature review. Int. J. Food Sci. Nutr..

[19] Kelleher B.P., Scott J.M., O’Broin S.D. (1990). Cryo-preservation of Lactobacillus leichmannii for vitamin B12 microbiological assay. Med. Lab. Sci..

[20] Alcock S.C., Finglas P.M., Morgan M.R.A. (1992). Production and purification of an R-protein-enzyme conjugate for use in a microtitration plate protein-binding assay for vitamin B12 in fortified food. Food Chem..

[21] Frenkel E.P., Kitchens R.L., Prough R. (1979). High-performance liquid chromatographic separation of cobalamins. J. Chromatogr..

[22] Honda K., Imanishi M., Takeda T., Kimura M. (2001). Determination of Vitamin B12 in Serum by HPLC/ICP-MS. Anal. Sci..

[23] Wang M., Schuster K., Asam S., Rychlik M. (2023). Challenges in the determination of total vitamin B12 by cyanidation conversion: Insights from stable isotope dilution assays. Anal. Bioanal. Chem..

[24] Huang B., Zhang J., Wang M., Cai Z. (2022). Determination of Vitamin B12 in Milk and Dairy Products by Isotope-Dilution Liquid Chromatography Tandem Mass Spectrometry. J. Food Qual..

[25] Ren X.N., Yin S.A., Yang Z.Y., Yang X.G., Shao B., Ren Y.P., Zhang J. (2015). Application of UPLC-MS/MS Method for Analyzing B-vitamins in Human Milk. Biomed. Environ. Sci..

[26] Zia H., Fischbach N., Hofsommer M., Slatnar A. (2023). Development and validation of HPLC-MS/MS method for simultaneous analysis of B vitamins present naturally or after fortification in fruit juices. LWT.

[27] Jiang X., Wang Y., Liu J. (2022). Simultaneous determination of four cobalamins in rat plasma using online solid phase extraction coupled to high performance liquid chromatography-tandem mass spectrometry: Application to pentylenetetrazole-induced seizures in Sprague-Dawley rats. PLoS ONE.

[28] Qiu X., Zhang H., Yin Y., Brandes H., Marsala T., Stenerson K., Cramer H., You H. (2019). Determination of active vitamin B12 (cobalamin) in dietary supplements and ingredients by reversed-phase liquid chromatography: Single-laboratory validation. Food Chem..

[29] Yang Y., Zhou B., Zheng C. (2024). The Fast Quantification of Vitamin B12 in Milk Powder by High-Performance Liquid Chromatography-Inductively Coupled Plasma Mass Spectrometry. Molecules.

[30] Santos F., Vera J.L., Lamosa P., de Valdez G.F., de Vos W.M., Santos H., Sesma F., Hugenholtz J. (2007). Pseudovitamin B12 is the corrinoid produced by Lactobacillus reuteri CRL1098 under anaerobic conditions. FEBS Lett..

[31] van den Oever S.P., Mayer H.K. (2022). Biologically active or just “pseudo”-vitamin B12 as predominant form in algae-based nutritional supplements?. J. Food Compos. Anal..

[32] Edelmann M., Aalto S., Chamlagain B., Kariluoto S., Piironen V. (2019). Riboflavin, niacin, folate and vitamin B12 in commercial microalgae powders. J. Food Compos. Anal..

[33] Temova Rakuša Ž., Roškar R., Hickey N., Geremia S. (2022). Vitamin B(12) in Foods, Food Supplements, and Medicines-A Review of Its Role and Properties with a Focus on Its Stability. Molecules.

[34] Shell T.A., Lawrence D.S. (2011). A New Trick (Hydroxyl Radical Generation) for an Old Vitamin (B12). J. Am. Chem. Soc..

[35] Padmanabhan S., Jost M., Drennan C.L., Elías-Arnanz M. (2017). A New Facet of Vitamin B12: Gene Regulation by Cobalamin-Based Photoreceptors. Annu. Rev. Biochem..

[36] Jost M., Simpson J.H., Drennan C.L. (2015). The Transcription Factor CarH Safeguards Use of Adenosylcobalamin as a Light Sensor by Altering the Photolysis Products. Biochemistry.

[37] Hogenkamp H.P.C. (1963). A Cyclic Nucleoside Derived from Coenzyme B12. J. Biol. Chem..

[38] Law P.Y., Wood J.M. (1973). The photolysis of 5′-deoxyadenosylcobalamin under anaerobic conditions. Biochim. Biophys. Acta (BBA)—Nucleic Acids Protein Synth..

[39] Schwartz P.A., Frey P.A. (2007). 5‘-Peroxyadenosine and 5‘-Peroxyadenosylcobalamin as Intermediates in the Aerobic Photolysis of Adenosylcobalamin. Biochemistry.

[40] Juzeniene A., Nizauskaite Z. (2013). Photodegradation of cobalamins in aqueous solutions and in human blood. J. Photochem. Photobiol. B Biol..

[41] Sohail M., Rakha A., Butt M.S., Iqbal M.J., Rashid S. (2017). Rice bran nutraceutics: A comprehensive review. Crit. Rev. Food Sci. Nutr..

[42] Xie C., Coda R., Chamlagain B., Edelmann M., Varmanen P., Piironen V., Katina K. (2021). Fermentation of cereal, pseudo-cereal and legume materials with *Propionibacterium freudenreichii* and *Levilactobacillus brevis* for vitamin B12 fortification. LWT.

[43] Marley E.C., Mackay E., Young G. (2009). Characterisation of vitamin B12 immunoaffinity columns and method development for determination of vitamin B12 in a range of foods, juices and pharmaceutical products using immunoaffinity clean-up and high performance liquid chromatography with UV detection. Food Addit. Contam. Part A.

[44] Moore S.J., Lawrence A.D., Biedendieck R., Deery E., Frank S., Howard M.J., Rigby S.E.J., Warren M.J. (2013). Elucidation of the anaerobic pathway for the corrin component of cobalamin (vitamin B_12_). Proc. Natl. Acad. Sci. USA.

[45] Xie C., Coda R., Chamlagain B., Edelmann M., Deptula P., Varmanen P., Piironen V., Katina K. (2018). In situfortification of vitamin B12 in wheat flour and wheat bran by fermentation with *Propionibacterium freudenreichii*. J. Cereal Sci..

