# Supplementary material for: Simultaneous Determination of Three Active Forms of Vitamin B12 In Situ Produced During Fermentation by LC-MS/MS
Source: Foods. 2025 Jan 17;14(2):309. doi: 10.3390/foods14020309 (PMC11764900; doi:10.3390/foods14020309)
Supplement: Supplementary file 1 [file foods-14-00309-s001.zip › foods-3362884-supplementary.pdf]

Supplementary Data

Supple. Figure 1 Calibration curves of three VB12 forms

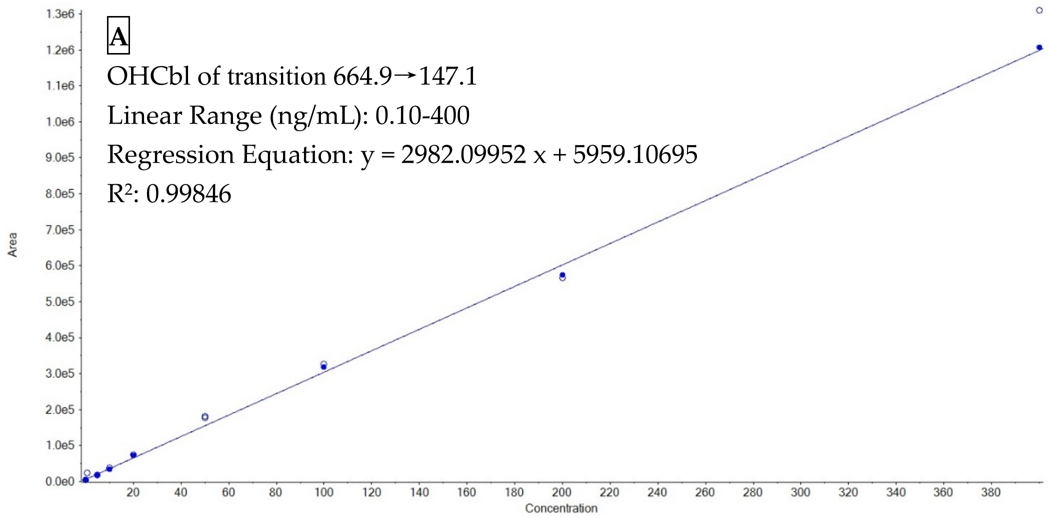

OHCbl of transition 664.9→147.1

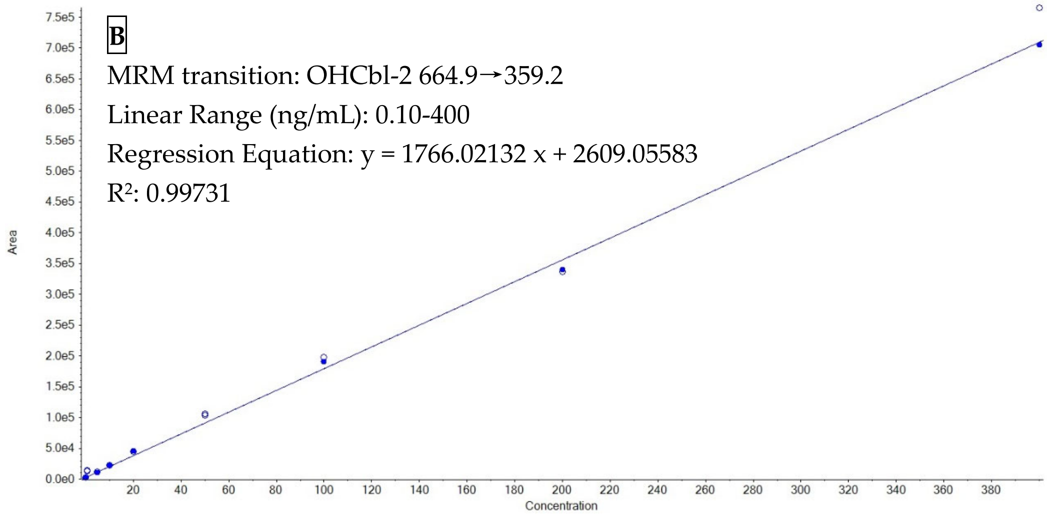

OHCbl of transition 664.9→359.2

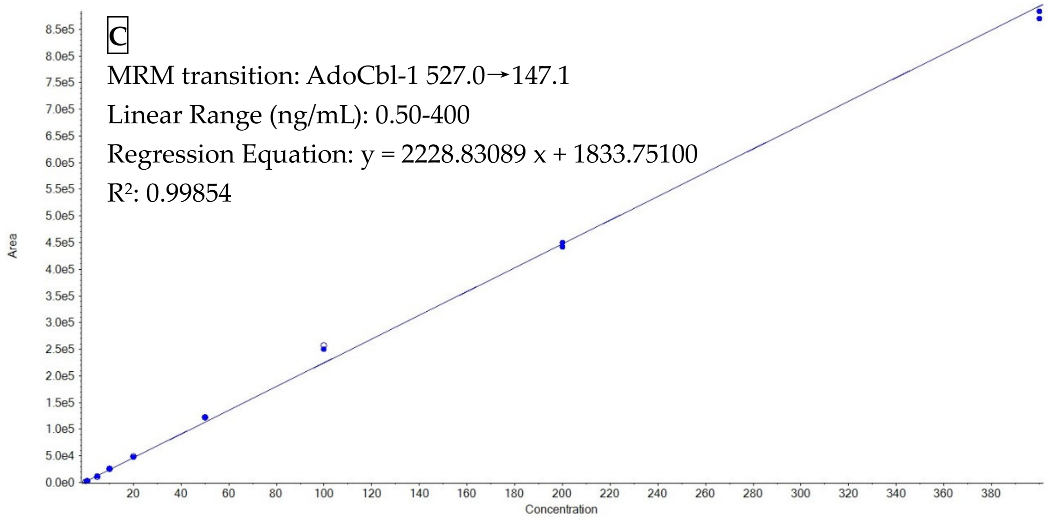

AdoCbl of transition 527.0→147.1

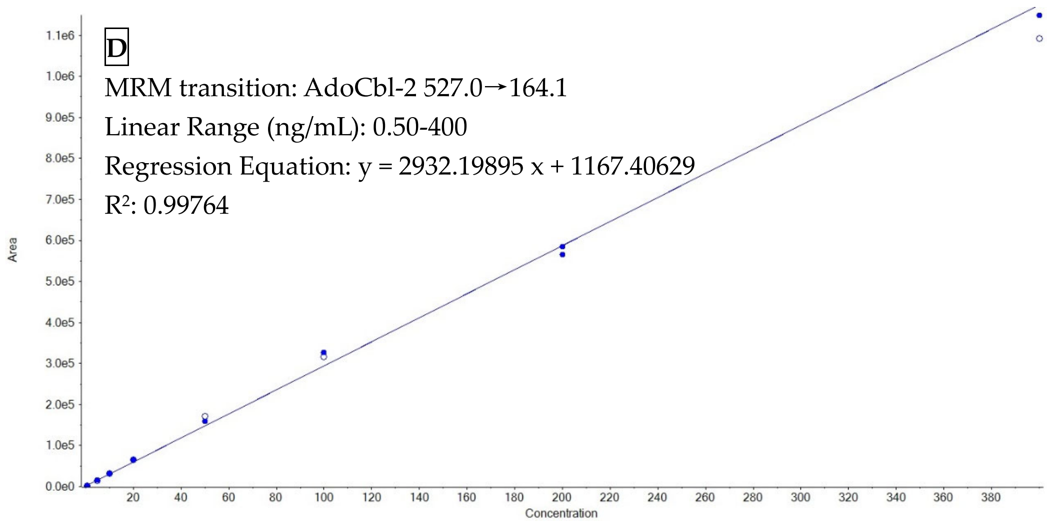

AdoCbl of transition 527.0→164.1

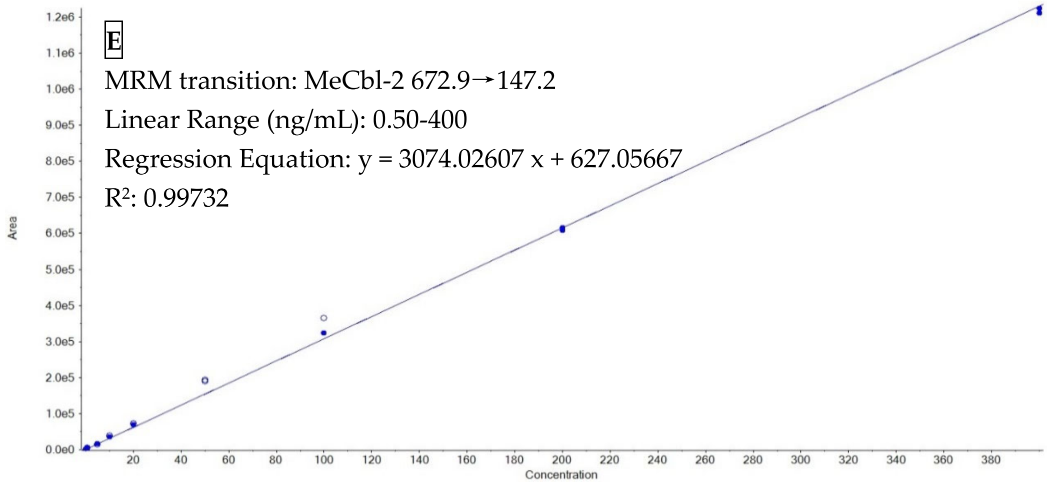

MeCbl of transition 672.9→147.2

Supple. Figure 2 Chromatographs of three VB12 standards in the lowest content

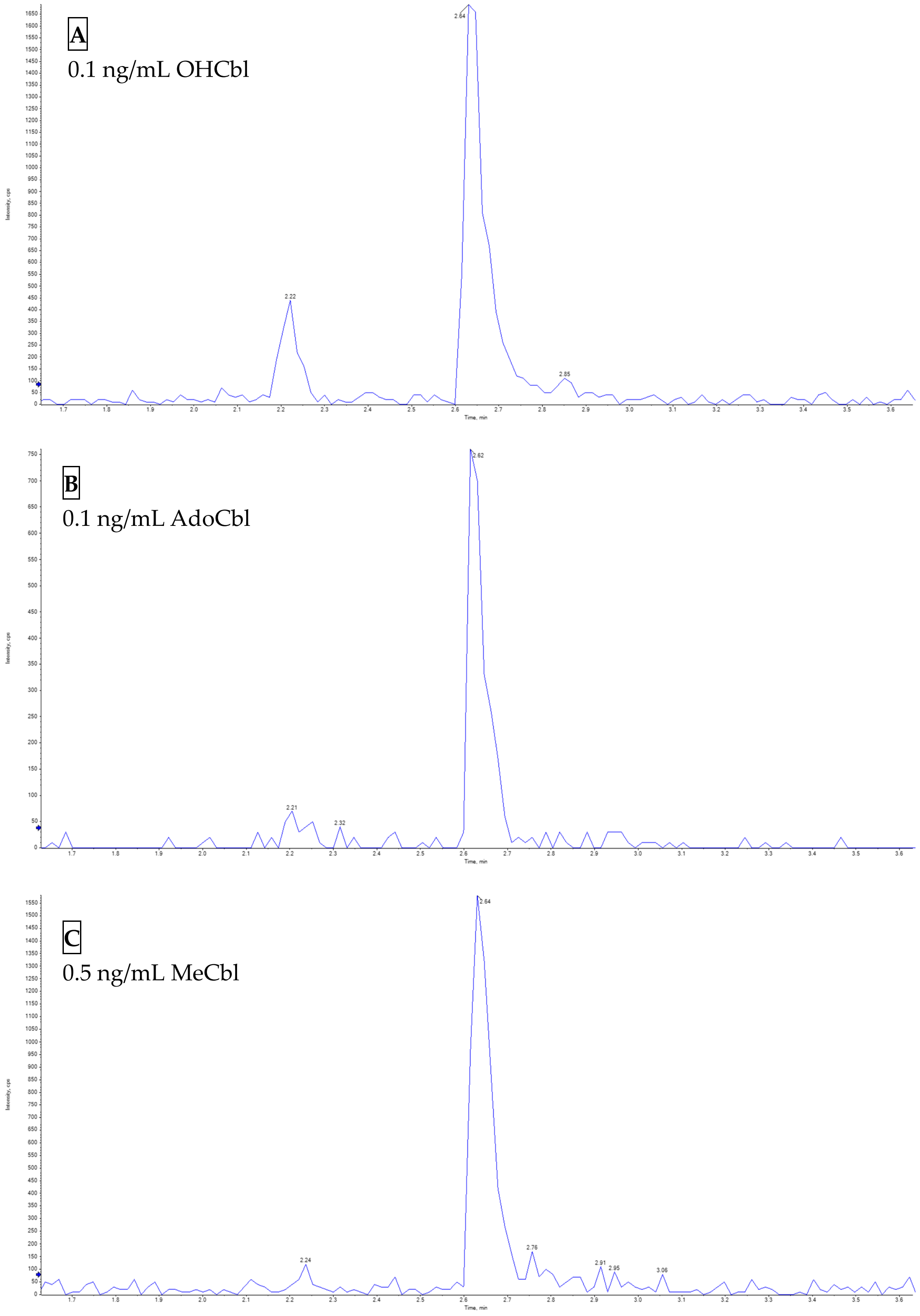

Supple. Table 1. The repeatability test of the method

	Time point 1	Time point 2	Time point 3
HOCbl	5.83%*	0.81%	3.62%
MeCbl	2.35%	4.69%	0.86%
AdoCbl	0.35%	0.15%	1.21%

* the relative standard deviation (RSD) of analysts on 3 time point with intervals longer than one week
